# Dataset of physicochemical, microbiological, and plant root parameters of 135 soils from various urban land uses Blois city, France

**DOI:** 10.1016/j.dib.2025.112265

**Published:** 2025-11-19

**Authors:** Marie-Paule Norini, Jennifer Harris, Sébastien Bonthoux, Cindy Arnoldi, Rachel Boscardin, Amélie Cantarel, Mickaël Charron, Thibauld Conte, Céline Cosson, Abigaïl Delort, Muriel Deparis, Jonathan Gervaix, Ulysse Guilloteau, Fadwa Khalfallah, Annie Millery, Agnès Richaume, Nathalie Tissot, Hafida Tris, Jean-Christophe Clément, Arnaud Foulquier, Catherine Joulian, Claude Le Milbeau, Xavier Le Roux, Emilie Lyautey, Mikael Motelica-Heino, Thomas Pommier, Nicolas Legay

**Affiliations:** a BRGM, F-45060, Orléans, France; b Institut des Sciences de la Terre d'Orléans (ISTO), UMR 7327, Univ. Orléans, CNRS, BRGM, OSUC, F-45071 Orléans, France; c INSA Centre Val de Loire, Université de Tours, CNRS, UMR 7324 CITERES, 37200, Tours, France; d LTSER, Zone Atelier Loire, France; e Univ. Grenoble Alpes, Univ. Savoie Mont Blanc, CNRS, LECA, Grenoble, France; f Université Lyon 1, Laboratoire d’Ecologie Microbienne LEM, INRAE (UMR 1418), CNRS (UMR 5557), Université de Lyon 1, VetAgroSup, 69622 Villeurbanne, France; g Université Savoie Mont Blanc, INRAE, CARRTEL, Thonon-Les-Bains, France; h UMR 6554 LETG, Rennes, France

**Keywords:** Urban land uses, Urban biodiversity, Soil microbiology, Enzymatic activities, Root functional traits, Soil characterization, Nitrogen cycle, CH_4_ production

## Abstract

If natural and cultivated soils have been widely investigated, urban soils are still poorly understood, especially in terms of the microbial diversity they harbor and its roles in providing soil functions and ecosystem services. This paper presents data collected from urban soils sampled at 135 sites from the medium-sized city of Blois (France), which correspond to different land uses randomly distributed in the city. In total, eight types of land use were identified, undergoing four levels of management intensity and positioned either in or out of the Loire floodplain. This data collection describes the main soil physicochemical characteristics (texture, pH, water status, chemical contents), plant traits (root functional traits) as well as abundances of broad taxonomic groups (bacterial, archaeal, and fungal), and of microbial functional groups involved in soil C, N, and P cycling, microbial diversity (sequencing of Bacteria, Archaea, and Fungi), and different microbial activities (respiration, activities linked to the nitrogen cycle, extracellular enzymatic activities, and potential methanogenesis). The dataset summarized in this article improves our knowledge about physicochemical and (micro)biological characteristics of urban soils and can be used as a reference for future studies of urban soils.

Specifications Table

**Table:** 

Subject	Soil science.
Specific subject area	Urban ecology, urban soil physicochemical parameters, urban plant functional traits, urban soil microbiology, urban soil enzymatic activities, urban soil nitrogen cycle.
Type of data	Tables, photos, maps, figures, and Microsoft®Excel files
Data collection	Soils were collected across the entire city of Blois to obtain soils from areas with contrasted land use (8 types) and management intensity (4 levels). Soils were collected in June 2020 from the topsoil (0–10 cm) of the identified sites. Composite soil samples (resulting from 9 soil cores) were obtained at each site, homogenized and sieved at 2 mm. Subsamples were stored at –20 °C (potential enzymatic activities, NEA, DEA, PNR, PNM, methanogenesis analysis, soil ammonium and nitrate concentration, DNA extraction and quantification of gene abundance) or 4 °C (soil moisture, soil respiration and elemental soil analysis). Soil subsamples were air-dried and ground (<80 µm) to measure soil pH, total soil C, and N. At each site, a tenth core 45 mm Ø (0–10 cm depth) was taken for root functional trait measurements. One extra core of 84 mm Ø (0–10 cm; 250cm3) was sampled next to the root core for measurement of bulk density and parameters related to soil water availability.
Data source location	Institution: ISTO, BRGM, CITERES, LECA, LEM, CARRTEL City/Town/Region: Blois Country: France Soil sampling site: Blois city, France (DMS coordinates: latitude (X): 47° 34′ 59.98″ and longitude (Y): 1° 19′ 59.99″)
Data accessibility	Repository name: https://data.indores.fr/ Data identification number: doi: 10.48579/PRO/MTZ0KB Direct URL to data: https://doi.org/10.48579/PRO/MTZ0KB The full database is accessible on a public repository The bacteria, archaea, and fungi sequence data are available on the European Nucleotide Archive system under project accession numbers PRJEB52287 (16S sequences) and PRJEB52222 (ITS2 sequences). The list of sequence accession numbers is also available in the full database https://doi.org/10.48579/PRO/MTZ0KB.
Related research article	

## 1 Value of the Data

• Presence of green areas in urban environments is an increasingly important factor for the preservation of biodiversity and ecosystem services in and around cities. This dataset provides useful information on microbial diversity and root functional traits in urban soils at the city scale. This aspect of urban biodiversity is scarcely studied, especially in France.
• Other researchers, policy makers or organizations interested in urban soil ecology can use the dataset presented here. In particular, improves our understanding on how land use types and/or soil management intensity drives urban soil microbial diversity and functioning.
• The data will be able to provide access to the existing relationships between urban microorganisms and their living environment.
• This dataset can be used to investigate and/or compare urban soil microbiological characteristics of Blois with other urban soils, for cities of different sizes.
• Data could be used in studies aiming to understand soil biodiversity determinism.

## 2 Background

Urbanization has profoundly disrupted global biogeochemical cycles, biodiversity, and ecosystem processes, making it one of the key sustainability challenges of the 21st century [1,2]. Urban-driven changes strongly modify soil abiotic conditions [3,4] through mechanisms such as pollution, water contamination, soil sealing, and the application of organic or inorganic fertilizers. These altered conditions directly affect essential soil biogeochemical functions—including nutrient cycling, carbon storage, and microbial activity [5]—while also indirectly reshaping microbial communities and their associated functions [6,7]

The aim of the study was to characterize the microbial diversity and activity of urban soils and to understand how these are influenced by soil physico-chemical parameters and green space management parameters in a variety of urban use contexts (e.g. collective housing, urban parks, sports fields, etc.).

The innovative nature of this project lies on the one hand in the ecosystem studied, i.e. urban soils. These soils are subject to strong anthropic pressure and management practices that can vary on very small scales. In public green spaces, these multiple management practices are strongly linked to site use and social factors (e.g. extensively managed parks for leisurely strolls, or lawns mown very frequently for the 'showcase' aspect of city centers). We still know very little about the diversity and functioning of soil microbial communities in these areas, particularly as their management varies enormously over fairly short time scales (annual to decadal).

The originality of our study also comes from the fact that it seeks to describe the intra-urban variability of green spaces induced by different uses and management, based on soil physico-chemical data, microbial community composition, microbial functional traits (gene abundance, enzymatic activities) and root traits.

## 3 Data Description

This article contains descriptive data (minimum, maximum, mean, standard deviation (n-1), and coefficient of variation (n-1)) for 24 abiotic and 32 biotic variables of 135 urban soil samples. The data are presented in tables either for the 135 sites or by classifying the data according to eight land uses.

Raw data were deposited in a public repository [data.InDoRES]. Microbial sequence data is available on the European Nucleotide Archive system under project accession numbers PRJEB52287 (16S sequences) and PRJEB52222 (ITS2 sequences).

The open-access research dataset is composed of one Excel file that contains two sheets: (i) one sheet with an explanation of the columns in the data table including a description of each variable, data type, and units of measurement and (ii) one sheet with the data table containing raw data. In addition to the parameters presented in this article, some data is detailed and only available in the Excel files in the public repository [data.InDoRES]: longitude, latitude, sampling date, current land-use, management intensity, sample location in or not in Loire flood plain, and percentages of sand, clay, and silt for each sample.

**Fig. 1:**
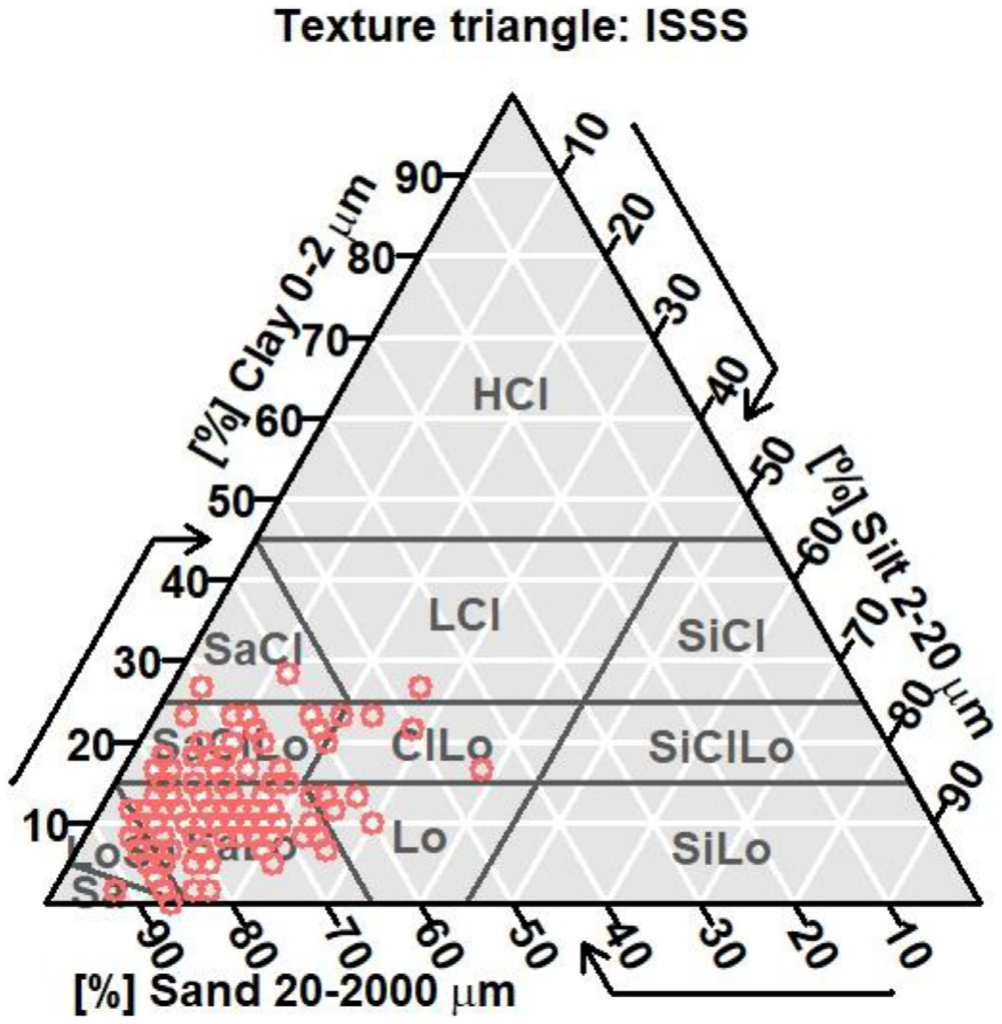
Texture distribution of the 135 soil samples.

**Fig. 2:**
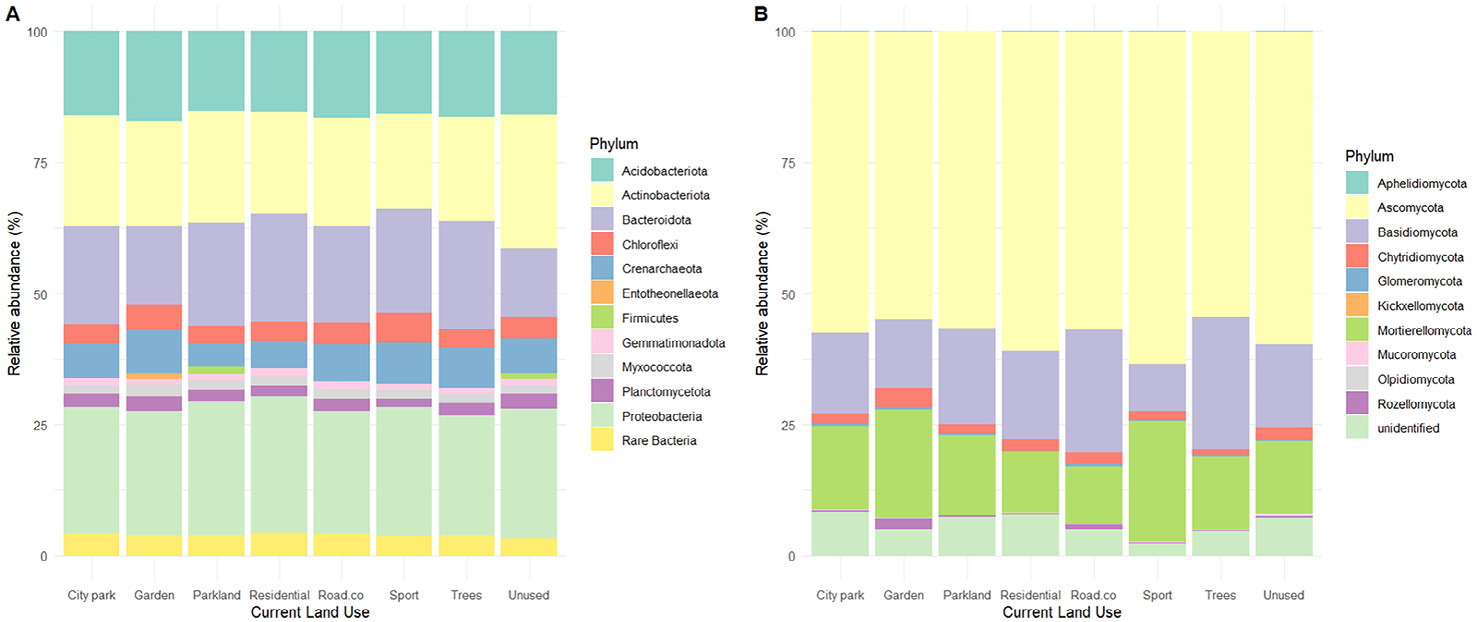
Relative abundance of (A) bacterial and (B) fungal compositions for the different land use. The rare bacteria represent the bacteria phylum with <1 % of relative abundance.

Table 1 describes the types of urban land use selected for soil sampling as well as the management intensity levels. Fig. 1 shows a map with the locations of the 135 soil samples selected among eight land uses in Blois. Fig. 2 shows the texture distribution of 135 soils.

**Table 1:** Description of urban land uses selected for soil sampling (*n* = number of sites).

Land use	Management intensity level	Soil modification level
**‘Parkland’**, Large natural public green spaces *n* = 10	1	Previously used for agricultural purposes (before 1970), low or moderate impact on soils
**‘Unused’**, Unused green areas, reserved for future buildings or flood absorption *n* = 27	2–3	Previously used for agricultural purposes (before 1970), low or moderate impact on soils
**‘Road.co’**, Road components (road verges, roundabouts, traffic islands) *n* = 29	1–4–3 high variability	Soils strongly modified during road construction
**‘Trees’**, Lines of trees associated to pedestrian paths *n* = 7	2–3	Soils modified during path construction
**‘City park’,** Green spaces with intermediate management intensity (small parks, squares) *n* = 16	2–3	Soils modified during park and square building
**‘Residential’**, Green spaces associated to large social housing *n* = 33	3	Soils modified (cutting and filling) during building and road construction (mainly in the 1970s)
**‘Sport’**, Sport fields *n* = 5	4	Highly modified soils with amendments and fertilizer inputs
**‘Garden’**, Highly maintained public gardens located in the historic old town *n* = 8	4	Highly modified soils with amendments and fertilizer inputs

Description of management intensity levels: (4) 18–25 grass mowings per year with residue export, lawn watering and scarification, fertilizer inputs, (3) 10–15 grass mowings per year, (2) 3–4 grass mowings per year, and (1) 1–2 grass mowings per year.

Tables include (i) the main physical soil properties (Table 2), (ii) the main chemical characteristics of soil samples (Table 3), (iii) parameters measured during the Rock Eval analysis (Table 4), (iv) the plant root functional traits (Table 5), (v) the information on microbial functional characteristics (Table 6), (vi) measures of microbial extracellular enzymatic activities (Table 7), (vii) data 16S and 18S rRNA gene abundances (Table 8), (viii) data on the abundances of genes involved in the nitrogen cycle (Table 9). and (ix) characteristics of PCR primer sets and of real-time quantitative PCR conditions used in this study (Table 10)

**Table 2:** Descriptive statistic of soil physical characteristics.

	Land use	n	Minimum	Maximum	Mean	SD	CV
**Soil water content** ( %)	**All land uses**	**135**	**4.93**	**44.63**	**19.10**	**8.31**	**0.43**
	Parkland	10	6.69	42.01	24.23	9.04	0.37
	Unused	27	4.93	33.26	10.34	6.17	0.60
	Road.co	29	7.70	31.94	19.56	6.98	0.36
	Trees	7	17.12	22.00	20.68	1.66	0.08
	City park	16	5.97	32.31	19.50	7.35	0.38
	Residential	33	8.04	33.29	21.59	7.06	0.33
	Sport	5	16.38	27.51	21.62	4.00	0.19
	Garden	8	13.87	44.63	26.57	9.42	0.35
**Gravimetric water content** (g_water_.g_dry soil_^−1^)	**All land uses**	**135**	**0.05**	**0.93**	**0.20**	**0.12**	**0.57**
	Parkland	10	0.07	0.42	0.24	0.09	0.37
	Unused	27	0.05	0.69	0.12	0.13	1.05
	Road.co	29	0.08	0.32	0.20	0.07	0.36
	Trees	7	0.17	0.22	0.21	0.02	0.08
	City park	16	0.06	0.32	0.19	0.07	0.38
	Residential	33	0.08	0.93	0.24	0.14	0.60
	Sport	5	0.20	0.54	0.29	0.14	0.48
	Garden	8	0.14	0.45	0.27	0.09	0.35
**Water filled pore space** ( %)	**All land uses**	**135**	**7.21**	**90.67**	**37.03**	**18.52**	**0.50**
	Parkland	10	9.48	58.83	40.42	14.17	0.35
	Unused	27	7.21	37.28	17.29	7.94	0.46
	Road.co	29	7.59	69.75	39.80	16.58	0.42
	Trees	7	31.72	88.80	45.74	19.52	0.43
	City park	16	12.24	81.59	35.83	14.99	0.42
	Residential	33	15.35	77.57	43.54	16.76	0.39
	Sport	5	26.10	75.23	54.36	19.56	0.36
	Garden	8	23.48	90.67	46.58	22.72	0.49
**Water holding capacity** ( %)	**All land uses**	**135**	**56.81**	**82.72**	**73.23**	**4.75**	**0.06**
	Parkland	10	68.96	80.35	73.91	4.13	0.06
	Unused	27	56.81	82.72	72.94	6.18	0.08
	Road.co	29	58.29	81.52	72.85	5.07	0.07
	Trees	7	71.13	79.23	74.97	2.69	0.04
	City park	16	66.23	78.26	73.20	3.31	0.05
	Residential	33	68.10	81.56	74.82	3.30	0.04
	Sport	5	63.00	77.37	70.14	5.30	0.08
	Garden	8	59.89	71.94	68.56	4.52	0.07
**Bulk density** (g_dry soil_.cm^−^³)	**All land uses**	**135**	**0.66**	**1.7**	**1.1**	**0.2**	**0.18**
	Parkland	10	0.78	1.19	1.02	0.13	0.13
	Unused	27	0.66	1.52	1.04	0.24	0.23
	Road.co	29	0.67	1.47	1.13	0.21	0.18
	Trees	7	0.98	1.60	1.17	0.20	0.17
	City park	16	0.80	1.38	1.09	0.19	0.18
	Residential	33	0.85	1.70	1.14	0.19	0.17
	Sport	5	1.00	1.50	1.26	0.18	0.14
	Garden	8	0.83	1.17	1.03	0.13	0.12
**Total porosity** ( %)	**All land uses**	**135**	**35.80**	**75.05**	**58.32**	**7.68**	**0.13**
	Parkland	10	55.15	70.53	61.39	5.08	0.08
	Unused	27	42.78	75.05	60.65	9.09	0.15
	Road.co	29	44.70	74.85	57.20	7.79	0.14
	Trees	7	39.60	63.12	55.81	7.63	0.14
	City park	16	48.09	69.92	58.81	7.34	0.12
	Residential	33	35.80	67.89	56.98	7.33	0.13
	Sport	5	43.54	62.45	52.51	6.78	0.13
	Garden	8	55.90	68.79	61.01	4.73	0.08

n: number of sites, SD: standard deviation (n-1), and CV: coefficient of variation (n-1).

**Table 3:** Descriptive statistic of soil chemical characteristics.

	Land use	n	Minimum	Maximum	Mean	SD	CV
**pH**	**All land uses**	**135**	**6.30**	**8.38**	**7.56**	**0.43**	**0.06**
	Parkland	10	6.81	8.38	7.65	0.46	0.06
	Unused	27	6.80	8.10	7.39	0.33	0.04
	Road.co	29	6.70	8.26	7.64	0.42	0.06
	Trees	7	7.33	8.16	7.80	0.31	0.04
	City park	16	7.05	8.12	7.64	0.32	0.04
	Residential	33	6.64	8.38	7.55	0.45	0.06
	Sport	5	6.30	7.30	6.76	0.42	0.06
	Garden	8	7.59	8.08	7.84	0.17	0.02
**Total soil carbon content** ( % of C)	**All land uses**	**135**	**0.93**	**7.53**	**3.14**	**1.26**	**0.40**
	Parkland	10	1.96	7.53	3.97	1.61	0.41
	Unused	27	1.03	5.99	2.54	1.11	0.44
	Road.co	29	1.62	6.95	3.23	0.98	0.30
	Trees	7	2.45	5.52	3.45	1.08	0.31
	City park	16	1.39	5.28	3.24	1.06	0.33
	Residential	33	0.93	6.27	2.80	1.08	0.39
	Sport	5	1.68	3.48	2.66	0.69	0.26
	Garden	8	3.03	7.34	5.08	1.49	0.29
**Total soil nitrogen content** ( % of N)	**All land uses**	**135**	**0.08**	**0.55**	**0.26**	**0.09**	**0.36**
	Parkland	10	0.18	0.46	0.30	0.09	0.29
	Unused	27	0.09	0.55	0.20	0.09	0.45
	Road.co	29	0.15	0.36	0.26	0.05	0.21
	Trees	7	0.21	0.34	0.26	0.04	0.17
	City park	16	0.11	0.37	0.27	0.07	0.27
	Residential	33	0.08	0.46	0.24	0.10	0.41
	Sport	5	0.17	0.35	0.26	0.07	0.28
	Garden	8	0.23	0.53	0.40	0.10	0.25
**NH_4_ soil content** (µg_N_.g_dry soil_^−1^)	**All land uses**	**135**	**1.70**	**22.57**	**10.09**	**4.48**	**0.44**
	Parkland	10	4.69	21.68	11.91	4.66	0.39
	Unused	27	1.70	22.57	8.05	4.57	0.57
	Road.co	29	1.72	20.80	10.59	4.58	0.43
	Trees	7	7.47	13.25	10.41	1.94	0.19
	City park	16	3.92	22.57	11.08	5.07	0.46
	Residential	33	1.81	19.70	10.25	4.62	0.45
	Sport	5	6.73	8.10	7.58	0.52	0.07
	Garden	8	7.81	14.68	11.61	2.61	0.23
**NO_3_ soil content** (µg_N_.g_dry soil_^−1^)	**All land uses**	**135**	**0.23**	**43.06**	**3.09**	**4.23**	**1.37**
	Parkland	10	0.42	6.06	1.77	1.97	1.12
	Unused	27	0.26	5.36	1.37	1.09	0.79
	Road.co	29	0.46	43.06	4.42	7.73	1.75
	Trees	7	0.65	4.69	1.76	1.42	0.81
	City park	16	0.23	6.04	2.63	1.56	0.59
	Residential	33	0.23	9.95	3.13	2.35	0.75
	Sport	5	1.22	5.93	4.04	1.98	0.49
	Garden	8	1.53	14.07	7.06	4.16	0.59
**PO_4_ soil content** (µg_PO4_.g_dry soil_-^1^)	**All land uses**	**135**	**0.00**	**30.04**	**4.02**	**6.62**	**1.65**
	Parkland	10	0.00	17.28	3.39	6.02	1.77
	Unused	27	0.00	26.13	8.17	8.52	1.04
	Road.co	29	0.00	30.04	3.28	6.98	2.13
	Trees	7	0.00	10.04	1.78	3.74	2.09
	City park	16	0.00	22.64	4.24	6.62	1.56
	Residential	33	0.00	9.32	1.64	2.80	1.70
	Sport	5	0.00	0.00	0.00	0.00	n.d.
	Garden	8	0.00	19.54	7.25	7.81	1.08

n: number of sites, SD: standard deviation (n-1), CV: coefficient of variation (n-1), and N.D. not determined.

**Table 4:** Descriptive statistic of soil Rock Eval parameters.

	Land use	n	Minimum	Maximum	Mean	SD	CV
**S1 signal** (mg_HC_.g_dry soil_^−1^)	**All land uses**	**135**	**0**	**0.45**	**0.01**	**0.04**	**2.96**
	Parkland	10	0.00	0.03	0.01	0.01	1.05
	Unused	27	0.00	0.01	0.00	0.01	1.06
	Road.co	29	0.00	0.06	0.01	0.01	1.27
	Trees	7	0.00	0.01	0.01	0.00	0.68
	City park	16	0.00	0.11	0.02	0.03	1.57
	Residential	33	0.00	0.45	0.03	0.08	2.81
	Sport	5	0.01	0.04	0.02	0.01	0.84
	Garden	8	0.01	0.01	0.01	0.00	0.00
**S2 signal** (mg_HC_.g_dry soil_^−1^)	**All land uses**	**135**	**0.08**	**15.17**	**5.32**	**2.31**	**0.43**
	Parkland	10	3.10	10.86	5.88	2.11	0.36
	Unused	27	0.08	10.23	4.76	2.39	0.50
	Road.co	29	1.74	8.46	5.24	1.80	0.34
	Trees	7	4.12	7.40	5.54	1.14	0.21
	City park	16	2.22	9.57	5.79	2.05	0.35
	Residential	33	0.13	9.74	4.53	2.11	0.47
	Sport	5	2.73	6.88	5.38	1.74	0.32
	Garden	8	5.03	15.17	8.87	3.17	0.36
**S3 signal** (mg_CO_.g_dry soil_^−1^)	**All land uses**	**135**	**1.60**	**13.44**	**5.39**	**1.84**	**0.34**
	Parkland	10	4.26	8.35	6.19	1.37	0.22
	Unused	27	1.99	9.06	4.65	1.75	0.38
	Road.co	29	1.62	8.68	5.49	1.44	0.26
	Trees	7	4.72	6.71	5.79	0.65	0.11
	City park	16	2.68	8.13	5.87	1.52	0.26
	Residential	33	1.60	9.24	4.88	1.84	0.38
	Sport	5	2.76	5.44	4.06	1.01	0.25
	Garden	8	4.66	13.44	8.12	2.59	0.32
**S3CO signal** **CO2 organic source** (mg_CO_.g_dry soil_^−1^)	**All land uses**	**135**	**0.45**	**3.01**	**1.44**	**0.41**	**0.29**
	Parkland	10	1.14	2.44	1.70	0.40	0.23
	Unused	27	0.60	2.22	1.24	0.39	0.32
	Road.co	29	0.45	2.31	1.44	0.36	0.25
	Trees	7	1.37	1.96	1.59	0.18	0.11
	City park	16	0.85	2.02	1.52	0.31	0.20
	Residential	33	0.57	2.04	1.37	0.44	0.32
	Sport	5	0.78	1.40	1.15	0.23	0.20
	Garden	8	1.46	3.01	1.92	0.49	0.26
**S3′CO signal** **CO organic and mineral source** (mg_CO_.g_dry soil_^−1^)	**All land uses**	**135**	**0.50**	**2.98**	**1.01**	**0.36**	**0.35**
	Parkland	10	0.60	1.74	1.16	0.35	0.30
	Unused	27	0.50	2.05	0.94	0.32	0.35
	Road.co	29	0.50	1.64	1.00	0.26	0.26
	Trees	7	0.81	1.33	1.00	0.16	0.16
	City park	16	0.50	1.84	1.11	0.34	0.30
	Residential	33	0.50	1.84	0.90	0.27	0.30
	Sport	5	0.70	0.81	0.75	0.06	0.08
	Garden	8	0.81	2.98	1.64	0.65	0.40
**S4CO signal** **CO organic source** (mg_CO_.g_dry soil_^−1^)	**All land uses**	**135**	**0.38**	**4.42**	**1.99**	**0.81**	**0.41**
	Parkland	10	1.07	2.94	1.87	0.66	0.35
	Unused	27	0.38	3.13	1.73	0.80	0.46
	Road.co	29	1.29	4.42	2.30	0.77	0.34
	Trees	7	1.60	2.80	2.17	0.43	0.20
	City park	16	0.53	3.04	1.82	0.65	0.36
	Residential	33	0.55	4.22	1.90	0.88	0.47
	Sport	5	0.51	3.06	2.16	1.09	0.51
	Garden	8	1.23	3.56	2.37	0.88	0.37
**S4CO2 signal** **CO_2_ organic source** (mg_CO2_.g_dry soil_^−1^)	**All land uses**	**135**	**15.62**	**156.14**	**61.85**	**20.30**	**0.33**
	Parkland	10	45.80	96.25	71.07	15.81	0.22
	Unused	27	15.62	106.25	54.60	20.25	0.37
	Road.co	29	38.58	91.18	63.44	13.56	0.21
	Trees	7	56.66	80.03	65.96	8.31	0.13
	City park	16	31.51	93.77	65.97	16.53	0.25
	Residential	33	20.03	98.72	55.21	18.98	0.34
	Sport	5	33.20	55.27	44.67	8.18	0.18
	Garden	8	58.03	156.14	95.28	29.89	0.31
**Pyrolysable organic carbon** (wt %)	**All land uses**	**135**	**0.14**	**1.82**	**0.67**	**0.26**	**0.38**
	Parkland	10	0.44	1.22	0.76	0.22	0.29
	Unused	27	0.23	1.24	0.60	0.26	0.44
	Road.co	29	0.22	1.01	0.67	0.20	0.29
	Trees	7	0.57	0.90	0.71	0.12	0.17
	City park	16	0.30	1.12	0.73	0.22	0.31
	Residential	33	0.14	1.20	0.59	0.24	0.41
	Sport	5	0.35	0.80	0.62	0.18	0.29
	Garden	8	0.63	1.82	1.08	0.36	0.34
**Residual organic carbon** (wt %)	**All land uses**	**135**	**0.44**	**4.39**	**1.77**	**0.57**	**0.32**
	Parkland	10	1.34	2.74	2.02	0.44	0.22
	Unused	27	0.44	3.00	1.56	0.58	0.37
	Road.co	29	1.14	2.67	1.83	0.39	0.21
	Trees	7	1.63	2.25	1.89	0.21	0.11
	City park	16	0.95	2.60	1.88	0.45	0.24
	Residential	33	0.58	2.79	1.59	0.55	0.34
	Sport	5	0.93	1.64	1.31	0.27	0.20
	Garden	8	1.67	4.39	2.70	0.83	0.31
**Total organic carbon** (wt %)	**All land uses**	**135**	**0.76**	**6.21**	**2.45**	**0.81**	**0.33**
	Parkland	10	1.78	3.64	2.77	0.64	0.23
	Unused	27	0.76	4.23	2.16	0.81	0.38
	Road.co	29	1.48	3.68	2.50	0.56	0.22
	Trees	7	2.21	3.15	2.60	0.33	0.13
	City park	16	1.25	3.71	2.61	0.65	0.25
	Residential	33	0.81	3.99	2.18	0.78	0.36
	Sport	5	1.28	2.44	1.93	0.44	0.23
	Garden	8	2.30	6.21	3.78	1.20	0.32
**Hydrogen index** (mg_HC_.g_TOC_^−1^)	**All land uses**	**135**	**3.59**	**318.08**	**213.29**	**45.07**	**0.21**
	Parkland	10	174.44	303.54	208.80	36.81	0.18
	Unused	27	3.59	317.84	217.63	55.17	0.25
	Road.co	29	80.08	276.40	206.34	45.66	0.22
	Trees	7	183.20	234.63	211.37	19.24	0.09
	City park	16	177.24	318.08	217.91	34.51	0.16
	Residential	33	13.69	246.69	201.78	46.19	0.23
	Sport	5	213.31	306.65	272.41	37.02	0.14
	Garden	8	208.18	251.27	232.50	14.27	0.06
**Oxygen index** (mg_CO2_.g_TOC_^−1^)	**All land uses**	**135**	**102.11**	**349.37**	**220.50**	**24.05**	**0.11**
	Parkland	10	202.87	239.81	224.06	11.54	0.05
	Unused	27	185.15	349.37	218.06	29.28	0.13
	Road.co	29	102.11	263.86	218.47	27.65	0.13
	Trees	7	212.61	242.75	223.31	10.48	0.05
	City park	16	203.53	248.73	224.48	13.59	0.06
	Residential	33	184.66	321.81	223.47	27.57	0.12
	Sport	5	200.11	223.40	209.81	9.80	0.05
	Garden	8	200.24	254.02	215.63	19.69	0.09

n: number of sites, SD: standard deviation (n-1), and CV: coefficient of variation (n-1).

**Table 5:** Descriptive statistic of plant roots characteristics.

	Land use	n	Minimum	Maximum	Mean	SD	CV
**Root diameter** (mm)	**All land uses**	**135**	**0.13**	**0.40**	**0.23**	**0.05**	**0.23**
	Parkland	10	0.15	0.30	0.20	0.04	0.22
	Unused	27	0.13	0.39	0.22	0.05	0.23
	Road.co	29	0.14	0.30	0.22	0.04	0.20
	Trees	7	0.18	0.30	0.22	0.04	0.17
	City park	16	0.13	0.38	0.23	0.06	0.28
	Residential	33	0.18	0.40	0.26	0.05	0.21
	Sport	5	0.19	0.29	0.23	0.04	0.19
	Garden	8	0.17	0.29	0.21	0.04	0.20
**Root dry matter content** (mg_dry root_ .g _fresh root_^−1^)	**All land uses**	**135**	**0.10**	**0.45**	**0.23**	**0.06**	**0.27**
	Parkland	10	0.11	0.30	0.21	0.06	0.30
	Unused	27	0.16	0.45	0.24	0.07	0.28
	Road.co	29	0.16	0.43	0.25	0.07	0.29
	Trees	7	0.15	0.31	0.21	0.05	0.26
	City park	16	0.17	0.33	0.23	0.05	0.22
	Residential	33	0.17	0.35	0.24	0.04	0.17
	Sport	5	0.10	0.21	0.13	0.05	0.35
	Garden	8	0.11	0.30	0.21	0.06	0.27
**Specific root length** (mg_root_.g _root dry mass_^−1^)	**All land uses**	**135**	**11.41**	**191.42**	**75.82**	**36.40**	**0.48**
	Parkland	10	47.75	149.02	93.11	36.60	0.39
	Unused	27	22.56	182.47	60.98	32.37	0.53
	Road.co	29	17.28	149.16	72.02	31.88	0.44
	Trees	7	29.89	112.52	67.90	32.11	0.47
	City park	16	11.41	140.90	73.57	39.79	0.54
	Residential	33	26.04	172.62	77.27	34.75	0.45
	Sport	5	41.13	130.27	91.40	32.42	0.35
	Garden	8	60.40	191.42	113.79	45.27	0.40
**Total root length** (m_root_)	**All land uses**	**135**	**104.41**	**2934.03**	**726.44**	**511.19**	**0.70**
	Parkland	10	278.11	1224.98	761.61	308.69	0.41
	Unused	27	163.30	2934.03	885.24	774.82	0.88
	Road.co	29	104.41	1809.69	737.75	468.44	0.63
	Trees	7	160.93	832.26	552.48	224.93	0.41
	City park	16	268.47	1580.95	766.06	443.05	0.58
	Residential	33	159.00	1863.83	524.38	364.10	0.69
	Sport	5	370.18	1548.97	924.13	445.09	0.48
	Garden	8	341.46	1485.29	888.52	452.88	0.51
**Root carbon content** ( % of C)	**All land uses**	**135**	**35.65**	**51.34**	**41.63**	**2.24**	**0.05**
	Parkland	10	35.65	44.14	40.65	2.24	0.05
	Unused	27	36.78	45.91	41.85	2.41	0.06
	Trees	7	40.00	42.50	41.34	1.01	0.02
	Road.co	29	36.15	51.34	41.62	2.70	0.06
	City park	16	37.22	45.30	41.14	1.83	0.04
	Residential	33	38.40	48.51	41.62	2.06	0.05
	Sport	5	39.60	44.31	42.34	1.86	0.04
	Garden	8	39.34	45.71	42.91	2.25	0.05
**Root nitrogen content** ( % of N)	**All land uses**	**135**	**0.25**	**4.09**	**0.75**	**0.39**	**0.52**
	Parkland	10	0.30	0.80	0.55	0.14	0.26
	Unused	27	0.25	1.16	0.57	0.19	0.33
	Road.co	29	0.34	4.09	0.81	0.66	0.81
	Trees	7	0.42	1.20	0.84	0.30	0.36
	City park	16	0.49	1.59	0.83	0.32	0.38
	Residential	33	0.34	1.58	0.81	0.30	0.36
	Sport	5	0.53	0.80	0.66	0.10	0.16
	Garden	8	0.64	1.20	0.91	0.21	0.23

n: number of sites, SD: standard deviation (n-1), and CV: coefficient of variation (n-1).

**Table 6:** Descriptive statistic results of soils microbial functional characteristics.

	Land use	n	Minimum	Maximum	Mean	SD	CV
**Basal respiration** (µg_CO2__—__C_ .g_dry soil_^−1^.h^−1^)	**All land uses**	**135**	**0.17**	**3.47**	**1.08**	**0.62**	**0.58**
	Parkland	10	0.37	2.56	1.26	0.58	0.46
	Unused	27	0.21	2.24	0.63	0.44	0.69
	Road.co	29	0.45	2.11	1.14	0.46	0.40
	Trees	7	0.17	1.59	1.14	0.48	0.42
	City park	16	0.36	2.05	0.97	0.54	0.55
	Residential	33	0.38	3.47	1.26	0.76	0.60
	Sport	5	1.76	2.74	2.10	0.39	0.18
	Garden	8	0.61	1.51	0.90	0.29	0.33
**Substrate (glucose) induced respiration** (µg_CO2__—__C_ .g_dry soil_^−1^.h^−1^)	**All land uses**	**135**	**0.10**	**8.38**	**2.80**	**1.86**	**0.67**
	Parkland	10	0.10	7.43	2.27	2.69	1.18
	Unused	27	0.10	4.77	2.23	1.23	0.55
	Road.co	29	0.10	6.72	2.46	1.83	0.74
	Trees	7	0.83	5.90	2.77	1.89	0.68
	City park	16	0.14	5.20	2.98	1.53	0.51
	Residential	33	0.17	8.38	3.34	1.98	0.59
	Sport	5	0.64	3.78	2.42	1.26	0.52
	Garden	8	0.21	8.16	4.22	2.31	0.55
**Potentially mineralizable nitrogen** (mg_N_.Kg_dry soil_^−1^.day^−1^)	**All land uses**	**135**	**−0.55**	**1.15**	**−0.03**	**0.19**	**−5.76**
	Parkland	10	−0.48	0.39	−0.10	0.24	−2.38
	Unused	27	−0.35	0.16	−0.05	0.09	−1.74
	Road.co	29	−0.55	1.15	−0.01	0.32	−36.29
	Trees	7	−0.23	0.04	−0.08	0.10	−1.22
	City park	16	−0.22	0.15	−0.04	0.10	−2.73
	Residential	33	−0.34	0.27	−0.02	0.16	−7.29
	Sport	5	−0.25	0.07	−0.07	0.14	−2.18
	Garden	8	−0.09	0.29	0.04	0.14	3.62
**Potentially nitrifiable nitrogen** (mg_N_.Kg_dry soil_^−1^.day^−1^)	**All land use**	**135**	**−3.75**	**8.26**	**0.74**	**1.73**	**2.33**
	Parkland	10	−0.35	6.96	0.90	2.39	2.66
	Unused	27	0.00	5.23	1.77	1.34	0.76
	Road.co	29	−1.28	8.26	0.61	1.87	3.05
	Trees	7	−0.86	0.42	0.06	0.43	7.58
	City park	16	−1.37	3.40	0.47	1.31	2.76
	Residential	33	−3.75	3.24	−0.27	1.15	−4.33
	Sport	5	1.97	4.15	3.01	0.94	0.31
	Garden	8	−1.02	4.92	1.58	2.27	1.43
**Denitrification enzyme activity** (µg_N_.h^−1^.g_dry soil_^−1^)	**All land uses**	**135**	**0.36**	**9.95**	**3.73**	**2.08**	**0.56**
	Parkland	10	1.67	6.15	4.06	1.54	0.38
	Unused	27	0.36	9.95	1.93	1.95	1.01
	Road.co	29	0.99	8.00	4.34	1.74	0.40
	Trees	7	4.84	6.37	5.71	0.61	0.11
	City park	16	0.50	6.33	3.70	1.65	0.45
	Residential	33	0.69	8.81	3.67	2.16	0.59
	Sport	5	4.91	8.30	6.31	1.44	0.23
	Garden	8	3.02	6.59	4.14	1.28	0.31
**Nitrification enzyme activity** (µg_N_.h^−1^.g_dry soil_^−1^)	**All land uses**	**135**	**0.00**	**2.65**	**0.75**	**0.54**	**0.72**
	Parkland	33	0.17	1.90	0.73	0.46	0.63
	Unused	29	0.14	1.40	0.78	0.33	0.42
	Road.co	27	0.04	2.65	0.43	0.50	1.17
	Trees	7	0.37	2.17	1.17	0.60	0.51
	City park	16	0.10	1.55	0.74	0.45	0.60
	Residential	10	0.28	1.14	0.55	0.26	0.48
	Sport	5	0.49	1.13	0.81	0.28	0.35
	Garden	8	0.00	2.46	1.67	0.88	0.53
**Methanogenesis activity** (nmol_CH4_.g_dry soil_^−1^.h^−1^)	**All land uses**	**135**	**0.00**	**21.00**	**0.16**	**1.81**	**11.62**
	Parkland	10	0.00	0.00	0.00	0.00	N.D.
	Unused	27	0.00	0.00	0.00	0.00	N.D.
	Road.co	29	0.00	21.00	0.72	3.90	5.39
	Trees	7	0.00	0.00	0.00	0.00	N.D.
	City park	16	0.00	0.00	0.00	0.00	N.D.
	Residential	33	0.00	0.00	0.00	0.00	N.D.
	Sport	5	0.00	0.00	0.00	0.00	N.D.
	Garden	8	0.00	0.00	0.00	0.00	N.D.

n: number of sites, SD: standard deviation (n-1), and CV: coefficient of variation (n-1). N.D. not determined.

**Table 7:** Descriptive statistic results of soil potential enzymatic activities.

	Land use	n	Minimum	Maximum	Mean	SD	CV
**Potential α-glucosidase activity** (nmol_AG_.g^−^_1dry soil_.h^−1^)	**All land uses**	**135**	**9.61**	**326.07**	**102.91**	**62.90**	**0.61**
	Parkland	10	15.05	221.48	103.28	62.95	0.61
	Unused	27	9.61	202.48	73.82	49.57	0.67
	Road.co	29	15.36	251.42	116.85	63.40	0.54
	Trees	7	66.60	215.00	120.95	60.52	0.50
	City park	16	17.24	207.73	111.60	49.87	0.45
	Residential	33	32.69	326.07	114.88	78.54	0.68
	Sport	5	25.00	98.94	58.46	29.86	0.51
	Garden	8	43.68	170.92	95.39	39.24	0.41
**Potential β−1,4-glucosidase activity** (nmol_BG_.g^−^_1dry soil_.h^−1^)	**All land uses**	**135**	**151.51**	**1428.87**	**574.73**	**236.56**	**0.41**
	Parkland	10	254.14	1024.65	611.63	248.84	0.41
	Unused	27	191.70	1082.95	546.66	236.88	0.43
	Road.co	29	151.51	1091.94	600.63	226.37	0.38
	Trees	7	329.68	1033.80	601.41	295.57	0.49
	City park	16	192.79	1010.21	613.85	202.20	0.33
	Residential	33	267.67	1428.87	617.40	256.10	0.41
	Sport	5	214.01	550.84	369.37	146.94	0.40
	Garden	8	231.46	519.48	380.12	98.22	0.26
**Potential β-d-cellobiosidase activity** (nmol_CB_.g^−^_1dry soil_.h^−1^)	**All land uses**	**135**	**8.06**	**293.95**	**104.62**	**59.82**	**0.57**
	Parkland	10	18.98	237.99	110.88	73.75	0.67
	Unused	27	8.06	224.53	83.97	57.49	0.68
	Road.co	29	16.22	238.63	119.12	60.01	0.50
	Trees	7	59.52	205.71	119.11	59.31	0.50
	City park	16	13.30	204.56	115.33	49.45	0.43
	Residential	33	32.28	293.95	112.49	65.42	0.58
	Sport	5	25.97	104.62	60.92	32.10	0.53
	Garden	8	35.99	123.11	74.62	26.36	0.35
**Potential β-xylosidase activity** (nmol_XYL_.g^−^_1dry soil_.h^−1^)	**All land uses**	**135**	**42.97**	**749.49**	**258.77**	**127.16**	**0.49**
	Parkland	10	66.07	411.17	250.09	104.16	0.42
	Unused	27	42.97	480.13	216.89	116.51	0.54
	Road.co	29	80.03	500.57	285.43	127.10	0.45
	Trees	7	160.00	479.81	280.51	136.09	0.49
	City park	16	54.18	468.69	286.17	107.63	0.38
	Residential	33	103.19	749.49	276.85	156.44	0.57
	Sport	5	109.76	240.01	163.34	59.31	0.36
	Garden	8	116.88	366.00	225.64	68.36	0.30
**Potential leucine aminopeptidase activity** (nmol_LAP_.g^−^_1dry soil_.h^−1^)	**All land uses**	**135**	**322.14**	**3203.89**	**1285.60**	**564.50**	**0.44**
	Parkland	10	556.15	2827.62	1297.46	765.38	0.59
	Unused	27	322.14	1619.54	932.34	354.37	0.38
	Road.co	29	433.94	2614.78	1405.90	573.27	0.41
	Trees	7	986.96	1917.82	1494.80	328.51	0.22
	City park	16	468.99	2090.61	1376.06	436.89	0.32
	Residential	33	480.26	2907.26	1277.13	565.00	0.44
	Sport	5	781.91	1428.69	1022.87	297.09	0.29
	Garden	8	1098.22	3203.89	1862.19	721.79	0.39
**Potential N-acetyl-β-glucosaminidase activity** (nmol_NAG_.g^−^_1dry soil_.h^−1^)	**All land uses**	**135**	**12.32**	**170.69**	**70.22**	**31.98**	**0.46**
	Parkland	10	40.25	167.87	89.33	44.95	0.50
	Unused	27	25.09	126.83	59.99	25.59	0.43
	Road.co	29	17.52	170.69	77.15	36.91	0.48
	Trees	7	46.13	80.55	60.46	13.07	0.22
	City park	16	12.32	132.64	68.80	29.26	0.43
	Residential	33	17.10	135.33	67.63	30.17	0.45
	Sport	5	56.94	120.23	80.92	26.25	0.32
	Garden	8	25.22	130.91	71.17	35.00	0.49
**Potential phosphatase activity** (nmol_PHOS_.g^−^_1dry soil_.h^−1^)	**All land uses**	**135**	**635.35**	**3670.48**	**1866.24**	**641.00**	**0.34**
	Parkland	10	635.35	3651.59	2033.17	988.33	0.49
	Unused	27	747.21	3041.00	1528.70	452.02	0.30
	Road.co	29	660.24	2787.52	1804.30	526.24	0.29
	Trees	7	1479.90	2498.44	1898.90	389.95	0.21
	City park	16	1118.83	3276.25	2053.86	606.82	0.30
	Residential	33	784.15	3043.60	1963.84	657.57	0.33
	Sport	5	2029.29	3648.05	2503.00	658.84	0.26
	Garden	8	1059.35	3670.48	1816.81	827.52	0.46

n: number of sites, SD: standard deviation (n-1), and CV: coefficient of variation (n-1).

**Table 8:** Descriptive statistic results of soil microbial abundance.

	Land use	n	Minimum	Maximum	Mean	SD	CV
**Amount of extracted soil DNA** (ng_DNA_.g_dry soil_^−1^)	**All land uses**	**135**	**5.02E+03**	**6.55E+04**	**2.30E+04**	**9190**	**0.4**
	Parkland	10	10,800	57,300	23,600	14,000	0.6
	Unused	27	5020	65,500	20,700	11,300	0.55
	Road.co	29	5930	38,100	23,500	7150	0.3
	Trees	7	18,100	40,400	26,700	7790	0.29
	City park	16	15,500	34,600	24,200	5820	0.24
	Residential	33	9520	35,500	20,100	7300	0.36
	Sport	5	17,300	34,000	24,600	6640	0.27
	Garden	8	23,500	47,700	34,000	8480	0.25
**Abundance of 16S rRNA gene** (16S gene copies.g_dry soil_^−1^)	**All land uses**	**135**	**9.27E+08**	**5.00E+10**	**1.18E+10**	**1.07E+10**	**0.91**
	Parkland	10	1.15E+09	3.2E+10	1.28E+10	1.21E+10	0.95
	Unused	27	1.63E+09	5E+10	1.23E+10	9.71E+09	0.79
	Road.co	29	927,000,000	3.91E+10	1.77E+10	1.11E+10	0.62
	Trees	7	3.57E+09	4.9E+10	2.28E+10	1.56E+10	0.68
	City park	16	2.36E+09	3.3E+10	1.18E+10	8.69E+09	0.74
	Residential	33	1.01E+09	3.1E+10	5.77E+09	7.45E+09	1.29
	Sport	5	2.9E+09	7.01E+09	4.58E+09	1.52E+09	0.33
	Garden	8	1.74E+09	2.16E+10	7.51E+09	6.4E+09	0.85
**Abundance of 18S rRNA gene** (18S gene copies.g_dry soil_^−1^)	**All land uses**	**135**	**1.4E+10**	**5.5E+10**	**2.18E+10**	**1.36E+10**	**0.63**
	Parkland	10	1.55E+10	1.07E+11	3.9E+10	2.7E+10	0.69
	Unused	27	3.94E+09	1.1E+11	3.48E+10	2.5E+10	0.72
	Road.co	29	4.63E+09	2.66E+11	5.15E+10	6.47E+10	1.26
	Trees	7	8.63E+09	6.55E+10	3.16E+10	2.17E+10	0.69
	City park	16	1.34E+10	1.52E+11	3.37E+10	3.37E+10	1
	Residential	33	7.31E+09	7.78E+10	2.69E+10	1.65E+10	0.62
	Sport	5	9.19E+09	4.06E+10	2.32E+10	1.17E+10	0.51
	Garden	8	1.4E+10	5.5E+10	2.18E+10	1.36E+10	0.63

n: number of sites, SD: standard deviation (n-1), and CV: coefficient of variation (n-1).

**Table 9:** Descriptive statistic of microbial functional genes.

	Land use	n	Minimum	Maximum	Mean	SD	CV
**Abundance of *nirS* gene** (*nirS* gene copies.g _dry soil_^−1^)	**All land uses**	**135**	**5.27E+05**	**1.87E+08**	**1.69E+07**	**2.04E+07**	**1.20**
	Parkland	10	1.03E+06	5.65E+07	1.57E+07	1.76E+07	1.13
	Unused	27	8.58E+05	8.98E+07	1.53E+07	1.84E+07	1.20
	Road.co	29	1.18E+06	3.97E+07	1.82E+07	1.11E+07	0.61
	Trees	7	5.09E+06	4.53E+07	2.48E+07	1.37E+07	0.55
	City park	16	3.58E+06	3.49E+07	1.34E+07	8.98E+06	0.67
	Residential	33	5.27E+05	4.80E+07	9.02E+06	1.11E+07	1.23
	Sport	5	5.11E+06	4.10E+07	2.63E+07	1.37E+07	0.52
	Garden	8	1.36E+07	1.87E+08	4.63E+07	5.79E+07	1.25
**Abundance of *nirK* gene** (*nirK* gene copies.g _dry soil_^−1^)	**All land uses**	**135**	**8.46E+07**	**4.70E+09**	**1.04E+09**	**8.36E+08**	**0.80**
	Parkland	10	1.30E+08	4.19E+09	1.17E+09	1.36E+09	1.15
	Unused	27	2.42E+08	4.70E+09	1.22E+09	9.61E+08	0.79
	Road.co	29	8.76E+07	2.76E+09	1.22E+09	7.24E+08	0.59
	Trees	7	6.34E+08	2.37E+09	1.32E+09	5.74E+08	0.43
	City park	16	3.69E+08	2.09E+09	1.14E+09	5.66E+08	0.50
	Residential	33	8.46E+07	2.84E+09	6.30E+08	6.02E+08	0.96
	Sport	5	3.77E+08	8.47E+08	5.53E+08	1.84E+08	0.33
	Garden	8	3.79E+08	4.12E+09	1.22E+09	1.21E+09	0.99
**Abundance of *nosZI* gene** (*nosZI* gene copies.g _dry soil_^−1^)	**All land uses**	**135**	**7.16E+05**	**3.68E+07**	**7.66E+06**	**5.88E+06**	**0.77**
	Parkland	10	1.68E+06	2.52E+07	7.57E+06	7.75E+06	1.02
	Unused	27	7.16E+05	3.68E+07	7.66E+06	7.13E+06	0.93
	Road.co	29	1.11E+06	2.29E+07	9.75E+06	5.86E+06	0.60
	Trees	7	5.43E+06	2.30E+07	1.03E+07	6.33E+06	0.61
	City park	16	3.39E+06	1.37E+07	7.23E+06	2.71E+06	0.37
	Residential	33	1.01E+06	2.25E+07	5.23E+06	4.44E+06	0.85
	Sport	5	2.93E+06	1.06E+07	6.38E+06	3.05E+06	0.48
	Garden	8	4.12E+06	2.69E+07	9.46E+06	7.41E+06	0.78
**Abundance of *nosZII* gene** (*nosZII* gene copies.g _dry soil_^−1^)	**All land uses**	**135**	**2.73E+04**	**4.12E+07**	**7.65E+06**	**9.21E+06**	**1.20**
	Parkland	10	9.55E+04	1.41E+07	5.13E+06	4.97E+06	0.97
	Unused	27	2.73E+04	1.58E+07	1.84E+06	3.29E+06	1.79
	Road.co	29	2.98E+05	1.37E+07	3.11E+06	3.09E+06	0.99
	Trees	7	7.90E+05	1.72E+07	5.03E+06	5.66E+06	1.13
	City park	16	9.11E+05	3.58E+07	1.00E+07	1.21E+07	1.21
	Residential	33	5.54E+05	2.24E+07	9.91E+06	7.12E+06	0.72
	Sport	5	1.33E+07	3.31E+07	2.26E+07	7.86E+06	0.35
	Garden	8	7.87E+06	4.12E+07	2.57E+07	1.01E+07	0.39
**Abundance of *nxrA* gene** (*nxrA* gene copies.g _dry soil_^−1^)	**All land uses**	**135**	**4.59E+03**	**2.95E+07**	**4.37E+05**	**2.68E+06**	**6.14**
	Parkland	10	9.94E+03	2.60E+05	1.21E+05	9.22E+04	0.76
	Unused	27	9.37E+03	1.05E+07	5.63E+05	2.00E+06	3.56
	Road.co	29	1.02E+04	2.95E+07	1.16E+06	5.45E+06	4.69
	Trees	7	1.65E+04	6.18E+05	1.62E+05	2.08E+05	1.28
	City park	16	1.00E+04	3.55E+05	7.49E+04	8.43E+04	1.13
	Residential	33	4.59E+03	1.99E+06	1.62E+05	3.49E+05	2.15
	Sport	5	7.46E+03	1.84E+05	6.44E+04	7.59E+04	1.18
	Garden	8	2.46E+04	5.67E+05	1.14E+05	1.84E+05	1.61
**Abundance of *NS* gene** (*NS* gene copies.g _dry soil_^−1^)	**All land uses**	**135**	**1.02E+07**	**5.12E+08**	**9.47E+07**	**7.46E+07**	**0.79**
	Parkland	10	2.35E+07	4.58E+08	1.07E+08	1.39E+08	1.30
	Unused	27	1.02E+07	5.12E+08	8.58E+07	9.33E+07	1.09
	Road.co	29	2.07E+07	2.06E+08	8.63E+07	4.20E+07	0.49
	Trees	7	5.42E+07	1.88E+08	1.08E+08	4.74E+07	0.44
	City park	16	4.20E+07	1.53E+08	9.15E+07	2.93E+07	0.32
	Residential	33	1.02E+07	2.12E+08	6.89E+07	4.45E+07	0.65
	Sport	5	5.37E+07	2.22E+08	1.43E+08	6.21E+07	0.43
	Garden	8	1.30E+08	3.51E+08	2.10E+08	7.83E+07	0.37
**Abundance of *amoA* gene** (*amoA* gene copies.g _dry soil_^−1^)	Parkland	10	9.88E+07	7.00E+08	2.38E+08	1.81E+08	0.76
	Unused	27	8.48E+05	2.03E+09	3.39E+08	3.98E+08	1.17
	Road.co	29	4.60E+07	8.03E+08	4.19E+08	2.29E+08	0.55
	Trees	7	1.07E+08	1.08E+09	6.17E+08	3.45E+08	0.56
	City park	16	1.16E+08	9.26E+08	4.38E+08	2.19E+08	0.50
	Residential	33	1.52E+07	8.30E+08	3.05E+08	1.99E+08	0.65
	Sport	5	2.93E+08	6.24E+08	4.48E+08	1.41E+08	0.32
	Garden	8	3.38E+08	1.49E+09	8.47E+08	4.18E+08	0.49
	Parkland	10	9.88E+07	7.00E+08	2.38E+08	1.81E+08	0.76
**Abundance of *amoB* gene** (*amoB* gene copies.g _dry soil_^−1^)	**All land uses**	**135**	**1.43E+06**	**2.15E+08**	**1.82E+07**	**2.09E+07**	**1.15**
	Parkland	10	3.39E+06	3.15E+07	1.43E+07	9.61E+06	0.67
	Unused	27	1.43E+06	6.91E+07	1.44E+07	1.49E+07	1.03
	Road.co	29	3.09E+06	2.15E+08	2.37E+07	3.85E+07	1.63
	Trees	7	8.39E+06	5.84E+07	2.43E+07	1.67E+07	0.69
	City park	16	8.03E+06	2.24E+07	1.46E+07	4.33E+06	0.30
	Residential	33	1.84E+06	4.59E+07	1.44E+07	9.81E+06	0.68
	Sport	5	1.88E+07	3.58E+07	2.69E+07	6.12E+06	0.23
	Garden	8	1.20E+07	5.60E+07	2.77E+07	1.43E+07	0.52
**Abundance of *mcrA* gene** (*mcrA* gene copies.g _dry soil_^−1^)	**All land uses**	**132**	**0.00E+00**	**1.49E+05**	**2.40E+04**	**2.75E+04**	**1.14**
	Parkland	10	0.00E+00	7.30E+04	2.03E+04	2.92E+04	1.44
	Unused	25	3.85E+02	8.14E+04	1.26E+04	1.86E+04	1.48
	Road.co	29	2.84E+02	9.15E+04	1.51E+04	2.49E+04	1.64
	Trees	7	4.80E+03	7.49E+04	1.96E+04	2.48E+04	1.27
	City park	15	0.00E+00	4.62E+04	1.27E+04	1.32E+04	1.04
	Residential	33	4.28E+03	1.49E+05	4.09E+04	2.75E+04	0.67
	Sport	5	4.83E+04	9.34E+04	6.64E+04	1.95E+04	0.29
	Garden	8	0.00E+00	1.06E+05	2.60E+04	3.39E+04	1.30
**Abundance of *pmoA* gene** (pmo*A* gene copies.g _dry soil_^−1^)	**All land uses**	131	6.81E+04	7.77E+06	1.45E+06	1.15E+06	0.79
	Parkland	9	4.26E+05	2.57E+06	1.25E+06	6.64E+05	0.53
	Unused	26	7.46E+05	4.00E+06	1.57E+06	9.15E+05	0.58
	Road.co	29	3.60E+05	4.16E+06	1.86E+06	8.59E+05	0.44
	Trees	7	8.94E+05	3.38E+06	2.05E+06	8.39E+05	0.41
	City park	16	3.26E+05	5.27E+06	1.34E+06	1.25E+06	0.94
	Residential	31	6.81E+04	1.42E+06	6.93E+05	3.19E+05	0.46
	Sport	5	2.87E+05	7.77E+06	3.20E+06	3.44E+06	1.07
	Garden	8	3.77E+05	1.72E+06	9.33E+05	4.64E+05	0.50

n: number of sites, SD: standard deviation (n-1), and CV: coefficient of variation (n-1).

The characterization of the main physical soil properties, including texture, bulk density, water content, and water holding capacity, is presented in Table 2. Texture distribution of the 135 samples is described in Fig. 1.

HCl: heavy clay; SaCl: sandy clay; SaClLo: sandy clay loam; SaLo: sandy loam; LoSa: loamy sand; Sa: sand; LCl: light clay; ClLo: clay loam; Lo: loam; SiCl: silty clay; SiClLo: silty clay loam; SiLo: silt loam

The characterization of the chemical properties of the soils, including pH, electrical conductivity, total carbon, total nitrogen, phosphorus, and other elemental concentrations, is provided in Table 3.

The quantification of soil organic matter content and thermal properties obtained through Rock Eval analysis, including total organic carbon, hydrogen and oxygen indices, and stability parameters, is reported in Table 4.

The measurement of plant root functional traits, such as root length, diameter, density, and specific surface area, is presented in Table 5.

The characterization of microbial functional characteristics is summarized in Table 6, while the measurement of extracellular enzymatic activities involved in organic matter decomposition and nutrient cycling is reported in Table 7.

The quantification of 16S and 18S rRNA gene abundances as indicators of bacterial/archaeal and eukaryotic biomass is presented in Table 8. The quantification of the abundances of genes involved in the nitrogen cycle, including genes associated with nitrification, denitrification, and nitrogen fixation, is reported in Table 9. The microbial composition of the bacterial and fungal, identified using Illumina sequencing are represented in the Fig 2

## 4 Experimental Design, Materials and Methods

### 4.1 Study site

Soil samples were collected in the city of Blois (46,660 inhabitants in 2020) located in central-western France. The mean annual temperature is 11.6 °C and annual rainfall is 640 mm. The city of Blois is situated on the bank of the Loire River, a river that has been little developed and canalized compared to other large rivers in Western Europe. The historic city center developed mainly during the Middle Ages and the Renaissance periods. A strong demographic growth took place after World War II between 1950 and 1980 (from 28,000 to 49,000 inhabitants) with the construction of residential blocks in the northwest of the city.

The urban uses and management practices of green spaces are diversified according to the location in the city and the types of urban forms (e.g. historic city center, collective residential areas, recreational and sports areas, industrial areas).

The 135 soil samples were collected from different types of land use in public areas. In total, eight types of land use were identified, undergoing four levels of management and positioned either in or out of the Loire flood plain (Fig. 1 and Table 1). The four levels of management are defined as follows:

Level 1: grass mowing 1–2 times per year,
Level 2: grass mowing 3–4 times per year,
Level 3: grass mowing 10–15 times per year,
Level 4: grass mowing 18–25 times per year with residue export, lawn watering and scarification, and fertilizer inputs.

Information on management modalities was collected from the Blois city municipality services.

### 4.2 Soil sample collection

**Fig. 3:**
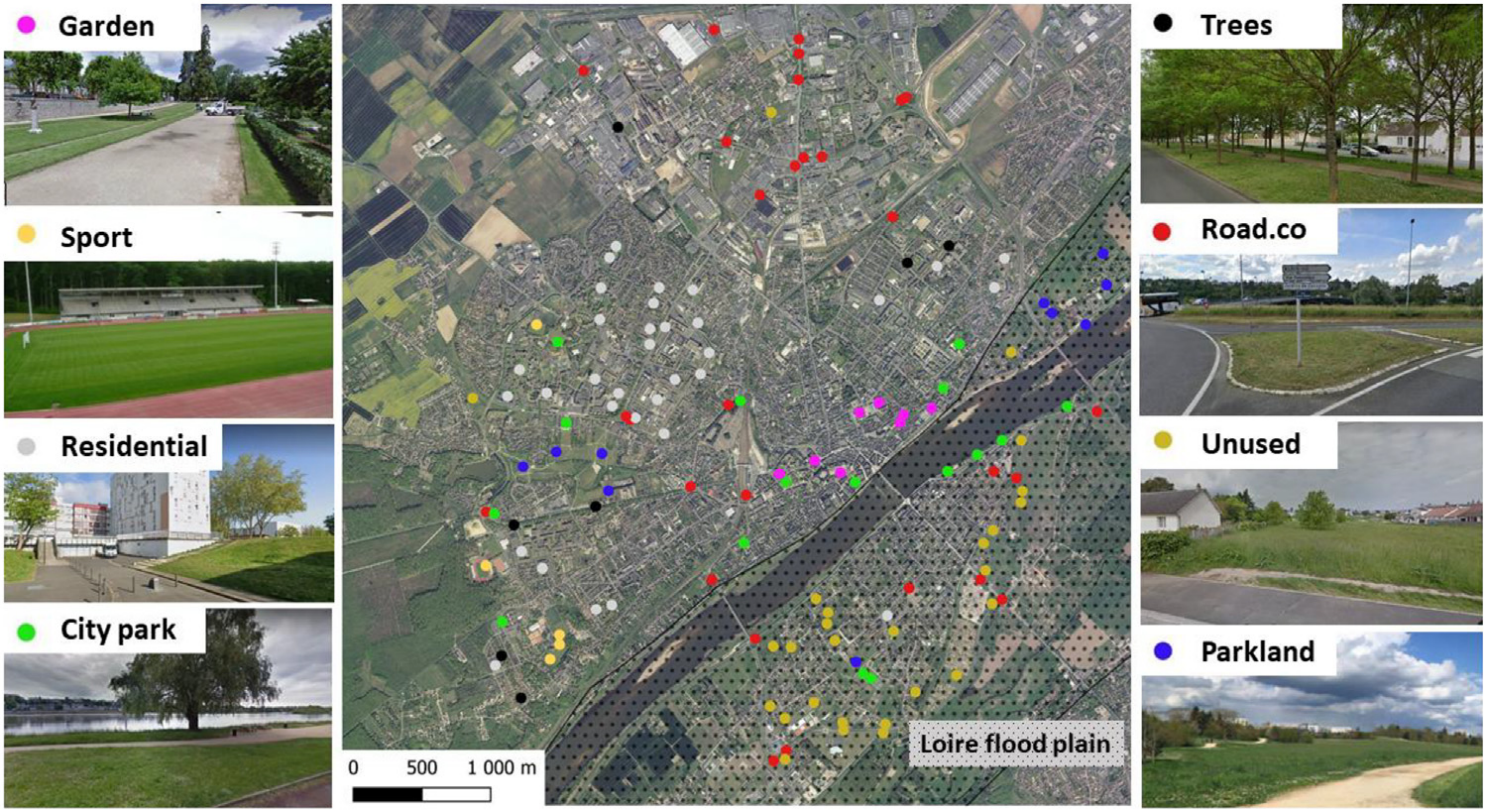
Location of the 135 soil samples selected among eight land uses in Blois. Description of land uses, and associated management intensity levels are presented in Table 1 (Photos, S. Bonthoux, 2019).

Soil samples were collected in June 2020 in 135 sites distributed among eight land uses in Blois city (Fig.3). The location of each site was pre-determined on a map with GIS and then accurately on the field and recorded using GPS. All coordinates are available in Excel file in the public repository [data.InDoRES].

At each site, nine soil cores were collected using an auger of 45 mm Ø at a depth of 0–10 cm. Soil cores were taken in a 3 × 3 m square, with one sample taken every metre or every metre on a 9-metre line, depending on the site. These nine cores were pooled into composite samples. A tenth core was also taken for root functional trait measurements. One extra core of 84 mm Ø (250cm^3^) was sampled next to the root core for measurement of bulk density and parameters related to soil water availability.

The 135 fresh composite soil samples were sieved through a 2 mm mesh. Subsamples were stored at –20 °C for further determination of extracellular enzymatic activities [8], soil ammonium and nitrate concentration, DNA extraction and quantification of 16S, 18S rRNA and functional gene abundances.The remaining subsamples were stored at 4 °C for immediate processing (within 72 h) for the determination of soil moisture parameters, potential enzymatic activities, potentially mineralizable nitrogen (PMN) and potentially nitrifiable nitrogen (PNN), potential denitrification enzyme activity (DEA), potential nitrification enzyme activity (NEA), methanogenesis and soil respiration (microbial basal and substrate-induced respiration). Later, the subsamples stored at 4 °C were processed to measure soil texture, bulk density, total soil porosity, pH, and Flash and RockEval soil elemental analysis. Roots were sampled from the dedicated soil core from each site, then carefully washed and stored at 4 °C in an alcohol solution, prior to analysis.

### 4.3 Physicochemical soil properties

Soil texture (sand, silt and clay fraction determination) was measured using a soil texture test kit (Model 1067; LaMotte, Chestertown, MD, USA) using the general method of mechanical analysis or soil separation. Percentages of sand, clay, and silt for each sample are available in the Excel files in the public repository [data.InDoRES].

Physical characteristics of soil samples (Table 2) integrate parameters related to soil water availability. Soil subsamples were dried at 105 °C for 48 h to determine soil water content (SWC). Gravimetric water content was determined by measuring fresh mass before water addition and dry mass of soil cores. Bulk density and total soil porosity were obtained by measuring the dry mass of a fixed-volume (250cm^3^) soil core. Prior to drying, 100 mL distilled water was added to saturate each soil core to calculate water holding capacity (WHC) and water filled pore space (WFPS).

Chemical characteristics of soil samples are presented in Table 3. Soil pH was measured in a 1:4 (soil/distilled water) solution with a pH electrode HI11310 (Hanna Instruments, Lingolsheim, France).

Soil subsamples were air dried and ground to a fine powder (<80 µm) to measure total soil C and N contents with a FlashEA 1112 elemental analyzer (Fisher Scientific Inc., Waltham, MA, USA) and using aspartic acid (Fisher Scientific Inc., Waltham, MA, USA) as the standard for the benchmark range with helium as the carrier gas. Flash pyrolysis involves heating the sample to very high temperatures (900 °C) in the absence of oxygen to determine the total hydrogen, carbon (C), nitrogen (N) and sulfur content in a soil sample using a thermal conductivity detector (TCD). The test dose is 1 to 5 mg.

Soil nitrate (NO_3_^−^), ammonium (NH_4_^+^) and orthophosphate (PO_4_^3−^) were extracted from 10 g of fresh soil with 45 ml of a 0.5 M K_2_SO_4_ solution. The N and phosphorus (P) concentrations were then measured on an automated photometric analyzer using standard colorimetric methods (Gallery Plus: Thermo Fisher Scientific, Waltham, Massachusetts, USA).

Ground (< 80 µm) soil samples were also used to measure total organic carbon (TOC), hydrogen index (HI) and oxygen index (OI) by Rock-Eval pyrolysis (Rock-Eval 6 Turbo, Vinci Technologies) (Table 4). Rock-Eval pyrolysis is a pyrolysis method in an open environment that allows rapid characterization of organic materials. The sample (30 to 100 mg) is pyrolyzed under an inert nitrogen atmosphere between 200 °C and 650 °C at increasing temperatures (+30 °C per minute). The effluents produced during this first step are continuously quantified by a flame ionization detector (FID) for hydrocarbon compounds (or HC hydrocarbons) and by two infrared cells for oxygen compounds (CO and CO_2_). In a second step, the sample passes through an oxidation oven for oxidation of the pyrolysis residue under an oxygenated atmosphere (air) between 400 and 700 °C at increasing temperatures (+30 °C per minute). The CO and CO_2_ emitted are again continuously detected by the infrared cells.

### 4.4 Plant root characteristics

Plant root characteristics are presented in Table 5. Roots were sampled from the dedicated soil core in each site. Soil cores were carefully washed in tepid water to allow separation of roots by floatation using sieve stacks with different mesh, namely 5.6 mm, 2 mm and 0.2 mm. Roots were placed into an alcohol solution (ethanol 10 %, acetic acid 5 %, v/v) and stored at 4 °C to maintain freshness until root morphology measurements using digital scanning. Before analysis, roots were suspended in 1 cm of demineralized water in a 29 × 42 cm clear acrylic tray and scanned at 600 dp.i. with an Epson LA2400 flatbed scanner. Each digital root image was processed using WINRHIZO software (Regent Instruments Inc., Sainte-Foy-Sillery-Cap-Rouge, Canada) to determine total root length and average root diameter. Roots were then weighed, dried at 70 °C and reweighed to calculate root dry matter content (RDMC) and specific root length (SRL). Finally, dry roots were ground to a fine powder (< 5 µm) for analysis of N and C concentrations with a FlashEA 1112 elemental analyzer (Fisher Scientific Inc., Waltham, MA, USA).

### 4.5 Microbial parameters

Description of microbial parameters is provided in Tables 6, 7, 8, and 9. The soil respiration system MicroResp™ (James Hutton Ltd., Aberdeen, United Kingdom) (Table 6) was used to measure microbial basal (BR) and glucose-induced respiration (substrate induced respiration, SIR). This colorimetric method is based on the detection of released CO_2_ in a 96-well plate by a gel containing a coloured indicator (cresol red) that changes colour from purple to yellow when in contact with CO_2_. The protocol used is that of the manufacturer. The CO_2_ emission by soil microorganisms was estimated by colorimetry at the absorbance wavelength 570 nm with an Omega SPECTROstar microplate spectrophotometer (BMG Labtech, Ortenberg, Germany) after a 6 h dark incubation period at 25 °C. The CO_2_ production rate is calculated following manufacturer’s instructions by converting the 6 h percentage of CO_2_ emitted to µg_C__—__CO2_.g_dry soil_^−1^.h^−1^.

Potentially mineralizable nitrogen (PMN) and potentially nitrifiable nitrogen (PNN) (Table 6) were determined on fresh soil samples using protocol taken and modified from previous studies [9]. Soil mineral nitrogen content at day 0 was determined by nitrate and ammonium extraction. Ten grams of equivalent dry weight (edw) of fresh soil was added to 45 mL 0.5 M K_2_SO_4_ solution, shaken at 2000 rpm for two minutes, and filtered through 0.2 mm acetate cellulose ClearLine® filters. Thirty to forty grams edw, adjusted to 60 % water holding capacity (WHC) using deionized water, was incubated in the dark at 20 °C for 3 weeks in 200 mL polystyrene pots. The pots were opened for a quarter of an hour twice a week to evacuate the accumulated CO_2_ and to renew the headspace atmosphere. The soils were mixed with a sterile spatula and then returned in a Plant Growth Chamber (MEMMERT HPP750 IPP PLUS) to incubation. At the end of the incubation nitrate and ammonium extractions were carried out as at days 0 with 10 g of dry soil. Ammonium was analyzed by colorimetry according to the NF ISO 15,923–1 (ISO, 2014) method with Thermo Fisher GalleryTM for ammonium (references 984,362 and 984,363) and nitrate were quantified by ionic chromatography (Dionex IC3000-SP-EG-DC system equipped with an AS50 autosampler and a conductimetric detector) according to the standard EN ISO 10,304–1 (ISO, 2009) method.

Potential denitrification enzyme activity (DEA) (Table 6) was measured according to previous study [10]. Briefly, about 10 g edw of fresh soil were placed at 28 °C under anaerobic conditions using a 90:10 He:C_2_H_2_ mixture to inhibit the N_2_O-reductase activity. Each flask was supplemented with 3 mL KNO_3_ (50 mg _N__—__NO3-._g_dry soi_l^−1^), glucose (0.5 mg_C_.g_dry soil_^−1^), and sodium glutamate (0.5mg_C_.g_dry soil_^−1^), completed with distilled water to reach the water-holding capacity. N_2_O concentration in the headspace was measured after 2 h, 3 h, 4 h, 5 h, and 6 h using a gas chromatograph (microGC R3000; SRA instruments, Marcy l’Etoile, France).

Potential nitrification enzyme activity (NEA) (Table 6) was determined according to previous study [11]. Briefly, 3 g edw from each fresh soil sample was supplied with a solution of (NH_4_)_2_SO_4_ (50µg_N__—__NH4+._g_dry soil_^−1^) and incubated under aerobic conditions (140 rpm, 28 °C, 10 h). Rates of NO_2_^−^ and NO_3_ production were quantified from the measurements made after 2 h, 4 h, 6 h, 8 h, and 10 h by colorimetric assessments using a Smartchem 200 (AMS Alliance, France).

Potential rates of methanogenesis were measured using 8 g edw of fresh soil according to protocols described by previous study [12] and adapted [13]. Soil was mixed with between 3.7 and 7.4 mL of distilled water depending on original soil moisture to achieve a theoretical soil moisture of 100 %, under aerobic conditions in 150 mL glass flasks with rubber stoppers. Incubation flasks were purged three times with He to achieve anaerobiosis, and the internal pressure was then adjusted to atmospheric pressure. All samples were incubated at 20 °C in the dark with gentle shaking. After 2 h, 4 h, and 18 h, headspaces were sampled and CH_4_ concentrations were analysed by gas chromatography (Agilent 490 MICRO GC, Agilent, Santa Clara, CA, USA). Methanogenesis activities are expressed as ng of CH_4_ per g of soil edw^−1^ h^−1^.

The potential activity of seven extracellular enzymes (α-Glucosidase, β−1,4-Glucosidase, β-d-cellobiosidase, β-Xylosidase, leucine aminopeptidase, N-acetyl-β-glucosaminidase, and phosphatase) are presented (Table 7). Enzymes activities were measured using standardized fluorimetric techniques [[14], [15]]. Fresh soil (2.75 g) was homogenized for 1 min in a Waring blender in 200 mL of a sodium acetate buffer solution adjusted to the mean pH of soil samples. The soil slurry (800 µL) was then added in technical duplicates to a 96-deep-well microplate with 200 µL of corresponding substrates at saturation concentration. Standard curves (0–100 µM concentration) were prepared in duplicates for each soil sample by mixing 800 µL of soil slurry with 200 µL of 4-methylumbelliferone (MUB) or 7-amino-4-methylcoumarin (MUC) in 96-deep-well microplates. Plates were incubated at 20 °C in the dark (3 h) on a rotary shaker (150 rpm) before centrifugation at 2900 g (3 min). The supernatant (250 µL) was transferred to a black Greiner flat-bottomed plate and fluorescence was measured on a microplate reader (Varioskan Flash, Thermo Scientific) with excitation wavelength set to 365 nm and emission set to 450 nm. After correcting for negative controls, potential enzyme activities were expressed as nmol.g_dry soil_^−1^.h^−1^.

Total soil DNA (Table 8) was extracted in duplicate from 0.5 g of wet weight of each soil sample, using the FastDNA® SPIN Kit for soil (MP Biomedicals, United States) per the manufacturer’s instructions with a FastPrep® −24 instrument (MP Biomedicals, United States) at a speed of 5 ms^−1^ for 30 s. The two replicates of each soil sample were pooled, and the extracted dsDNA was then quantified by the Quantifluor® dsDNA sample kit fluorimetry and a Quantus Fluorometer (Promega, United States) following the manufacturer’s protocol.

The abundances of the bacterial universal marker (16S rRNA gene) and fungal marker (18S rRNA gene) were assessed by real-time quantitative PCR (qPCR) in a CFX Connect (BioRad) with primer sets and thermocycling conditions explained in Table 10. Serial decimal dilutions of a linearized plasmid carrying the target gene (five-point for 16S rRNA of *P. putida* KT2440 and six-point for 18S rRNA gene of *F. oxysporum* DSM62267) were used to generate a linear calibration curve of threshold cycle versus a number of gene copies (ranging from 10^3^ to 10^7^ for *P. putida* KT2440 and from 10^2^ to 10^7^ for *F. oxysporum* DSM62267). Each DNA sample was analysed twice. Data were expressed as gene copy numbers per gram of dry weight soil.

**Table 10:** Characteristics of PCR primer sets and of real-time quantitative PCR conditions used in this study.

Target genes	Primer	Target	Base sequences 5′−3′	Size (bp)	Reaction mixture (20 µL reaction volumes)	Amplification program	Temps	Ref
*16S rRNA*	341F	Bacterial abundance	5′-CCTACGGGAGGCAGCAG-3′	175	–2 µl soil DNA extract (1 ng.µL^−1^) –10 µL of Sso advanced Supermix (Bio-Rad) –0.16 µL of each primer (50 ρmol.µL^−1^) –0.2 µL of T4 bacteriophage gene 32 Product (500 ng.µL^−1^) (MP Biomedicals)	–Denaturation: 95 °C/3 min –35 cycles: 95 °C/30 s; 60 °C/30 s; 72 °C/30 s; 80 °C/30 s (data acquisition) –Melting curve analysis: 0.5 °C/10 s from 65 °C to 95 °C	60	[15]
	515R		5′-ATTACCGCGGCTGCTGGCA-3′					
*18S rRNA*	FR1	Fungal abundance	5′-AICCATTCAATCGGTAIT-3′	390	–2 µl of soil DNA extract (1 ng.µL^−1^) –10 µL of Sso advanced Supermix (Bio-Rad) –0.2 µL of each primer (50 µmol.µL^−1^).	–Denaturation: 95 °C/5 min –40 cycles: 95 °C/15 s; 50 °C/30 s; 72 °C/30 s; 80 °C/30 s (data acquisition) –Melting curve analysis: 0.5 °C/10 s from 70 °C to 95 °C	50	[16]
	FF390		5′-CGATAACGAACGAGACCT-3′					
*nirS*	nirSCd3aF	abundance of denitrifying bacteria	5′-GTSAACGTSAAGGARACSGG-3′	422	- H_2_O qsp 25 µL - 1.875 µL of each primer (20 µM) - 0.5 µL of BSA (10 mg.mL^−1^) - 12.5 µL iTaq Universal SYBR Green Supermix (Bio-Rad) - 5 µL of soil DNA extract (2.5 ng.µL^−1^)	- Denaturation: 95 °C/3 min - 5 cycles: 95 °C/15 s; 59 °C/30s - 35 cycles: 95 °C/15 s; 54 °C/30 s;80 °C/5 s (data acquisition) - Melting curve analysis: 0.5 °C/0.05 s from 65 °C to 95 °C	54	[17]
	nirSR3cd		5′-GASTTCGGRTGSGTCTTGA-3′					
*nirK*	nirK876	abundance of denitrifying bacteria	5′-ATYGGCGGVCAYGGCGA-3′	164	- H_2_O qsp 20 µL - 1.25 µL of each primer (20 µM) - 0.4 µLof BSA (10 mg.mL^−1^) - 10 µL iTaq Universal SYBR Green Supermix (Bio-Rad) - 2 µL of soil DNA extract (2.5 ng.µL^−1^)	- Denaturation: 95 °C/3 min - 5 cycles: 95 °C/15 s; 63 °C/30s - 30 cycles: 95 °C/15 s; 58 °C/30 s;80 °C/5 s (data acquisition) - Melting curve analysis: 0.5 °C/0.05 s from 65 °C to 95 °C	58	[18]
	nirK1040		5′-GCCTCGATCAGRTTRTGGTT-3′					
*nosZI*	nosZ2F	abundance of denitrifying bacteria	5′-CGCRACGGCAASAAGGTSMSSGT-3′	267	- H_2_O qsp 25 µL - 1.875 µL of each primer (20 µM) - 0.5 µL of BSA (10 mg.mL^−1^) - 12.5 µL iTaq Universal SYBR Green Supermix (Bio-Rad) - 5 µL of soil DNA extract (2.5 ng.µL^−1^)	- Denaturation: 95 °C/3 min - 5 cycles: 95 °C/15 s; 65 °C/30s - 30 cycles: 95 °C/15 s; 60 °C/30 s;80 °C/5 s (data acquisition) - Melting curve analysis: 0.5 °C/0.05 s from 65 °C to 95 °C	60	[19]
	nosZ2R		5′-CAKRTGCAKSGCRTGGCAGAA-3′					
*nosZII*	nosZ-II-F	abundance of denitrifying bacteria	5′-CTI GGI CCI YTK CAY AC-3′	745	- H_2_O qsp 25 µL - 1.25 µL of each primer (20 µM) - 0.750 µL of BSA (10 mg.mL^−1^) - 12.5 µL iTaq Universal SYBR Green Supermix (Bio-Rad) - 8 µL of soil DNA extract (2.5 ng.µL^−1^)	-Denaturation: 95 °C/3 min - 40 cycles: 95 °C/15 s; 58 °C/60 s;80 °C/5 s (data acquisition) - Melting curve analysis: 0.5 °C/0.05 s from 65 °C to 95 °C	58	[20]
	nosZ-II-R		5′-GCI GAR CAR AAI TCB GTR C-3′					
*nxrA*	F1 NORA	abundance of nitrifying bacteria	5′-CAGACCGACGTGTGCGAAAG-3′	320	- H_2_O qsp 25 µL - 1.875 µL of each primer (10 µM) - 12.5 µLQuantiTect SybrGreen PCR Master Mix - 8 µL of soil DNA extract (2.5 ng.µL^−1^)	- Denaturation: 95 °C/15 min - 40 cycles: 95 °C/15 s; 55 °C/30 s;72 °C/30 s (data acquisition) - Melting curve analysis: 0.5 °C/0.05 s from 65 °C to 95 °C	55	[21]
	R2 NORA		5′-TCCACAAGGAACGGAAGGTC-3′					
*NS*	Ns675f	abundance of nitrifying bacteria	5′-GCGGTGAAATGCGTAGAKATCG-3′	71	- H_2_O qsp 25 µL - 2.5 µL of each primer (10 µM) - 0.5 µL of BSA (10 mg.mL^−1^) - 12,5 µL iTaq Universal SYBR Green Supermix (Bio-Rad) - 4 µL of soil DNA extract (2.5 ng.µL^−1^)	- Denaturation: 95 °C/3 min - 5 cycles: 95 °C/15 s; 66 °C/30 s; 72 °C/5sec - 35 cycles: 95 °C/15 s; 62 °C/30 s;72 °C/5 s (data acquisition) - Melting curve analysis: 0.5 °C/0,05 s from 65 °C to 95 °C	62	[22]
	Ns746r		5′-TCAGCGTCAGRWAYGTTCCAGAG-3′					
*amoA*	Crenamo A23f	abundance of nitrifying bacteria	5′-ATGGTCTGGCTWAGACG-3′	628	- H_2_O qsp 20 µL - 1 µL of primer F (20 µM) - 1.25 µL of primer R (20 µM) - 0.4 of BSA (10 mg.mL^−1^) - 10 µL iTaq Universal SYBR Green Supermix (Bio-Rad) - 4 µL of soil DNA extract (2,5 ng.µL^−1^)	- Denaturation: 95 °C/3 min - 40 cycles: 95 °C/15 s; 57 °C/45 s;72 °C/60 s (data acquisition) - Melting curve analysis: 0.5 °C/0.05 s from 65 °C to 95 °C	57	[23]
	Crenamo A616r		5′-GCCATCCABCKRTANGTCCA-3′					
*amoB*	amoA1F	abundance of nitrifying bacteria	5′-GGGGHTTYTACTGGTGGT-3′	491	- H_2_O qsp 20 µL - 2 µL of each primer (10 µM) - 0.4 of BSA (10 mg.mL^−1^) - 10 µL iTaq Universal SYBR Green Supermix (Bio-Rad) - 4 µL of soil DNA extract (2.5 ng.µL^−1^)	- Denaturation: 95 °C/3 min - 40 cycles: 95 °C/15 s; 54 °C/45 s;72 °C/5 s (data acquisition) - Melting curve analysis: 0.5 °C/0.05 s from 65 °C to 95 °C	54	[24]
	amoA2R		5′-CCCCTCKGSAAAGCCTTCTTC-3′					
*mcrA*	ME1	abundance of methanogenic archaea	5′-GCMATGCARATHGGWATGTC-3′	760	H2O qsp 25 µL - 1.25 µL of each primer (10 µM) - 2.5 µL of BSA (3 mg.mL^−1^) - 12.5 µL Brilliant III SYBR Master Mix (Agilent) - 5 µL of soil DNA extract (1 ng.µL^−1^)	- Denaturation: 95 °C/3 min - 45 cycles: 95 °C/5 s; 52 °C/15 s; 60 °C/20 s (data acquisition) - Melting curve analysis: 0.5 °C/0.05 s from 60 °C to 95 °C	52	[25]
	ME2		5′-TCATKGCRTAGTTDG GRTAGT-3′					
pmoA	pmoA189f	abundance of methanotrophic bacteria	5′-GGN GAC TGG GAC TTC TGG-3′	508	H2O qsp 25 µL - 1.25 µL of each primer (10 µM) - 2,5 µL of BSA (3 mg.mL^−1^) - 12.5 µL Brilliant III SYBR Master Mix (Agilent) - 5 µL of soil DNA extract (1 ng.µL^−1^)	Denaturation: 95 °C/10 min - 45 cycles: 94 °C/45 s; 60 °C/60 s; 72 °C/90 s (data acquisition) - Melting curve analysis: 0.5 °C/0.05 s from 60 °C to 95 °C	60	[25]
	mb661r		5′-CCG GMG CAA CGT CYT TAC C-3′					

'Ref' indicates the reference for each method, 'Temps' indicates the annealing temperature ( °C), and 'Size' indicates the amplicon size (bp).

Abundances of microbial functional groups involved in N cycling were quantified from the DNA extracted from each soil sample by measuring the gene copy numbers present per g of soil (expressed for dry soil) (Table 9). The abundances of ammonia-oxidizing bacteria (AOB) and archaea (AOA) were quantified based on the gene copy numbers present per g of dry soil for the ammonia monooxigenase gene *amoA* (for both bacterial and archaeal). The abundances of nitrite oxidizers (NOB) were quantified by targeting the abundances of the 16S rRNA gene specific to *Nitrospira* spp. and of the *nrxA* gene of *Nitrobacter* spp. Abundances of nitrite reducers were measured based on the copy numbers of the nitrite reductase genes *nirS* and *nirK*, while the abundances of N_2_O reducers were quantified by targeting the N_2_O reductase genes *nosZ1* and *nosZ2*. All primer pairs and related qPCR conditions are provided in Table 10. Quantitative real-time PCR (qPCR) assays were carried out using SYBR green as the fluorescent dye on a CFX Connect Real-Time PCR Detection System (Bio-Rad). Efficiencies of all performed qPCRs were in the range 85–99 %. R^2^ values for standard curves were always above 0.95. Possible inhibition of PCR was tested in advance and appropriate dilutions were chosen (data not shown). The abundances of methanogenic archaea and methanotrophic bacteria were quantified based on the copy numbers of the methyl coenzyme M reductase gene marker (*mcr*A gene) and of the particulate methane monooxygenase gene marker (*pmo*A gene), respectively. Assays were carried out by real-time quantitative PCR (qPCR) in an Agilent AriaMx Real-Time PCR System (Agilent Technologies) with primer sets and thermocycling conditions explained in Table 10. Serial decimal dilutions of a linearized plasmid carrying the target gene (seven-point, *mcr*A gene of *Methanosarcina thermophila* or *pmo*A gene of *Methylomonas methanica*) were used to generate a linear calibration curve of threshold cycle versus a number of gene copies (ranging from 10^1^ to 10^7^ copies). Each DNA sample was analysed twice. Data were expressed as gene copy numbers per gram of dry weight soil.

The diversity of the bacterial and fungal communities (Fig. 3) was determined by Illumina sequencing. PCR reactions of a portion of target genes were performed using the AccuStart II PCR ToughMix kit, followed by cleaning (HighPrep PCR beads, Mokascience). Universal primer sets 515WF/918WR [26] for the *16S rRNA* gene (V4-V5 region) and w404 and w40 [27] for the *ITS2* gene were used for amplifications. An Illumina MiSeq instrument at the genomics platform GeT-PlaGe (Auzeville, France) generated sequences. Sequence data are available on the European Nucleotide Archive system under project accession numbers PRJEB52287 (16S sequences) and PRJEB52222 (ITS2 sequences). The list of sequence accession numbers is available in the full database https://data.indores.fr/; https://doi.org/10.48579/PRO/MTZ0KB.

## Limitations

The study presents some methodological limitations. The uneven number of sites and partial geographic clustering across land uses is directly related to the difference in the areas covered by different land uses across the city and therefore representative of the diversity of areas covered by different land uses.

## Ethics Statement

Not applicable

## Credit Author Statement

Sébastien Bonthoux, Jean-Christophe Clément, Arnaud Foulquier, Jennifer Harris, Xavier Le Roux, Nicolas Legay, Emilie Lyautey, Mikael Motelica-Heino, Marie-Paule Norini, and Thomas Pommier wrote the initial project.

Sébastien Bonthoux and Nicolas Legay designed the sampling plan.

Sébastien Bonthoux, Muriel Deparis, Ulysse Guilloteau, Jennifer Harris and Nicolas Legay carried out the sampling campaign in the field.

Jennifer Harris, Marie-Paule Norini, Catherine Joulian, Hafida Tris, Mickaël Charron, Céline Cosson, and Thibauld Conte carried out the analyses and wrote the material and method part for the BRGM.

Jean-Christophe Clément, Emilie Lyautey, Annie Millery and Nathalie Tissot carried out the analyses and wrote the material and method part for the CARRTEL laboratory.

Sébastien Bonthoux, Muriel Deparis, Ulysse Guilloteau and Nicolas Legay carried out the analyses and wrote the material and method part for the CITERES laboratory.

Rachel Boscardin, Claude Le Milbeau, Mikael Motelica-Heino, and Marie-Paule Norini carried out the analyses and wrote the material and method part for the ISTO laboratory.

Cindy Arnoldy and Arnaud Foulquier carried out the analyses and wrote the material and method part for the LECA laboratory.

Amélie Cantarel, Abigaïl Delort, Jonathan Gervaix, Xavier Le Roux, and Thomas Pommier carried out the analyses and wrote the material and method part for the LEM laboratory.

Marie-Paule Norini performed the formal analysis and wrote the first version of the paper.

All authors reviewed the manuscript.

## Data Availability

Dataset of physicochemical, microbiological, and plant root parameters of 135 soils from various urban land uses Blois city, France (Original data).
